# Epidemiological differences of lower urinary tract symptoms among female subpopulations and group level interventions

**DOI:** 10.4103/0970-1591.44256

**Published:** 2008

**Authors:** Kameswararao Atchuta Avasarala, Syed Meraj Ahmed, Sujatha Nandagiri, Swati Tadisetty

**Affiliations:** Department of Community Medicine, Prathima Institute of Medical Sciences, Nagunur, Karimnagar-505 417, Andhra Pradesh, India

**Keywords:** Karimnagar district, lower urinary tract symptoms, working women, socioeconomic and quality of life losses

## Abstract

**Objectives:**

1) To study the risk factor profiles of lower urinary tract symptoms (LUTS) among adolescent girls, housewives and working women and its socioeconomic and quality of life losses. 2) To undertake risk factor modifications using the adolescent girls.

**Design and Setting:**

Cross-sectional descriptive study followed by educational intervention.

**Statistical Methods:**

Cluster sampling, Proportions, confidence intervals, Chi square and t-Tests and Logistic regression.

**Materials and Methods:**

House to house survey was done in two villages and one urban ward. Seventy-five housewives, 75 working women and 180 adolescent girls were asked about the risk factors and losses due to LUTS. Three teams of adolescent girls were utilized to bring about behavioral modifications. Impact was measured through user perspectives obtained from the participants.

**Results:**

Risk factors, social, economic and quality of life losses were different among the three female populations. Overall prevalence of LUTS among the three groups is 61(18.5%). Improper anal washing technique, malnutrition, presence of vaginal discharge, use of unsanitary menstrual pads, pinworm infestation and use of bad toilets were the significant causes among girls. Presence of sexually transmitted diseases was a contributing factor among housewives and working women. Prolonged sitting the posture was also contributing to LUTS among working women. Seventy-four per cent of beneficiaries expressed that intervention is useful.

**Conclusions:**

The causes for LUTS and their consequences were differing among the three female subpopulations. Specific group level interventions using trained girls were successful.

## INTRODUCTION

Several studies[1–5] revealed that Lower Urinary Tract Symptoms (LUTS) are common among females and more than in men.[4] The LUTS are known to cause socioeconomic as well as quality of life losses. Lowe NK in his study showed that the symptoms of vaginitis and LUTS affected military women′s quality of life.[6] The importance of assessment of quality of life losses was stressed by Espuna Pons in his study on LUTS.[7] Broseta Rico observed differences in the profiles of LUTS between menopausal and young women.[8] In this study, an attempt has been made to find out the epidemiological profles of LUTS among adolescent grls, housewives and working women with an intention to modify their negative risk factors/behaviors. Socioeconomic and quality of life losses were also elicited. Educational interventions were conducted for the girls, housewives and working women based on their risk factors.

## MATERIALS AND METHODS

This study included the survey and intervention phases. House to house survey was conducted in three selected clusters: two villages of Mothe and Anthergam and one urban ward of Kattarampur in Karimnagar district. One assistant professor from the department of Community medicine with the help of two lady doctors conducted the survey. These lady doctors were specially trained in the department for two days regarding interviewing the participants. The lady doctors interrogated 180 adolescent girls, 75 housewives and 75 working women. Questionnaires (see appendix) containing open-ended questions about age, occupation, literacy status, LUTS, type of the toilets used, anal washing technique after defecation, unsanitary pad usage during menses, history of pinworm infestation, presence of sexually transmitted diseases (STDs), usage of intrauterine contraceptive device (IUCD), loss of family life, loss of sexual life, loss of public relationships, loss of leisure opportunities, working hours, loss of physical independence, loss of wages, loss of leaves, reprimands from superiors or teachers etc were filled by the lady doctors. It was tested and retested in 10% of the study population and deficiencies were rectified. Survey was completed in 15 days.

- The following case definitions were utilized for the study.
  - LUTS considered were burning during micturition; Strangury; Pain during micturition; Increased Frequency of micturition; Urgency for micturition and Dull lower abdominal pain.[9]
  - Anal Washing technique: Direction from front to back (anus to behind) was considered as a right technique. Washing in the direction from anus to genitals was considered as wrong technique as there was a chance of soiling the genitals with feces and scope for urinary tract infection (UTI).
  - Malnutrition was defined as body mass index less than 19.
  - Syndromic approach of diagnosing STD under National AIDS Control Programme was utilized for the study.
  - Prolonged sitting was continuous sitting for more than 6 hours.
- Data was analyzed for all the variables of LUTS using SPSS version 16. Proportions, confidence intervals and X^2^ test and t-Tests were calculated. Contribution of the risk factors causing LUTS among females was analyzed using logistic regression models.

In the intervention phase, 60 adolescent school girls were selected to participate in the intervention. Girls were selected as they will have free time for follow-up. They were divided into three action teams according to their target group: adolescent girls′ team, housewives team and working women′s team. They were trained in the department using audiovisual aids for 10 days. This training was imparted in local vernacular using the lady doctors, faculty and health educators of the department. Teaching topics were selected based on the results of risk factors obtained through analysis. Training was conducted in two sessions: one general session for three days for all the 60 girls about the etiology, prevalence, prevention and management of LUTS in general. Later, the specific sessions for seven days, targeting each group with main emphasis on the risk factors found by analysis among each group were taken up. Topics on menstrual hygiene, sexually transmitted infections, anal washing technique, importance of sterile pad usage, good nutrition, care while using intrauterine devices etc were selected for behavioral modification. Adolescent girls were mainly targeted for menstrual hygiene improvement. In case of housewives, the stress was given on preventing STDs and safe sex practices. Working women were told about prevention of the occupational hazards. These points were stressed during training: Why do women have LUTS more often than men? What can I do if I have frequent LUTS? How serious are the LUTS? The following advices on preventing LUTS were given: drinking plenty of water to flush out bacteria, not holding urine, urinating when one feels to urinate, wiping from front to back after bowel movements, urinating after having sex, using enough lubricant during sex, avoiding using diaphragm if LUTS was present etc.

Pre-testing and post-testing was done to know the effect of training imparted. Impact of training to action teams was found significant in all the three teams (t_df19_=2.09, *P*<0.05 for girls’ motivating team, t_df19_=4.262, *P*< 0.05 for housewives’ motivating team and t_df19_=4.055, *P*<0.05 for working women motivating team). The trained girls were advised to motivate all the females in that group (not just the UTI sufferers) by persistent persuasive techniques. The idea was to carry out disease prevention for the whole group. They did it for a period of three months at the rate of 2 hours per week. The impact was evaluated through user perspectives of all the three groups. After three months of intervention, the adolescent girls, housewives and working women were asked to give their own opinion about the effects of intervention. They were asked to grade it as good, fair or poor according to their own impressions. Follow-up was done by the girls and the lady doctors. About 50% of them are still continuing the lifestyle modifications.

## RESULTS

Overall prevalence of LUTS in the study population was 61(18.5%). It is 23(13%), 22(29%) and 16(21%) among adolescent girls, housewives and working women respectively [Table 1]. As a whole, it is 21% in the poor, almost 79% in middle calss and rare in high-class females. LUTS profile in adolescent girls: Prevalence of LUTS was found to be more (17.8%) in girls who had attained menarche than those who hadn't (1.6%) (χ2=4.09, df1, CI=95%, *P*< 0.05). The prevalence of LUTS was significantly more in those girls using unsanitary pads during menstruation, practicing improper anal washing technique, having vaginal discharge, malnourished, having pinworms in stools and using bad toilets [Table 2]. Absence from classes of their schools, losing leisure opportunities and reprimands from teachers and parents were the main losses found in this adolescent girls' group. Twelve girls were absent for classes in the school. Eight expressed work loss while six complained of financial difficulties. Six girls are working as housemaids in the houses and as daily laborers. Their earnings are considered for calculating economic losses. LUTS profile among housewives: prevalence was more in the age group of 20-35 years, 14(63%) and declined with age to 9%. Mean duration of suffering from LUTS among housewives was one to five years.LUTS were seen more in middle class housewives. STDs, unsanitary pads’ usage, poor anal wash, vaginal discharge and malnutrition were observed as the significant causes [Table 3]. Family problems, loss of sexual life, loss of leisure opportunities, financial burden, and poor attention to children were the common losses experienced by housewives. LUTS profile among working women: using bad toilets, poor anal wash, STDs, prolonged sitting and vaginal discharge were the main reasons [Table 4]. Occupations with too much of sitting are more affected (42%). LUTS were more 14 (87.5%) in the first 10 years of employment and declined later. While 14 working women (87.5%) were suffering in the first 10 years of employment, only two (12.5%) were suffering later. Sickness absenteeism, loss of leisure opportunities, loss of sexual life, loss of wages and leaves, reprimands from superiors, and physical immobility were the main losses noticed among this group [Table 5]. Economic losses were: cumulative work days lost per year −110 days per year in this group. Total cumulative earnings lost by this group were 24500 rupees/year; total cumulative leaves lost by the group are 61 days in this year. LUTS were more in the first 20 years of married life and declined later in the housewives and working women. While 22 (57.9%) of housewives and 16 (21%) working women were suffering in the first 20 years of married life, only six(15.8%) were suffering later. About 74% of beneficiaries expressed that they were benefited by educational intervention. Impact of intervention is significant (X^2^_ df4_ = 14.98, *P*< 0.05,)

**Table 1:** Prevalence of lower urinary tract symptoms among study population

Population	LUTS Present (%)	LUTS Absent (%)
Adolescent girls N=180	23(13)	157(87)
Housewives N =75	22 (29)	53(71)
Working women N = 75	16(21)	59(79)
Total = 330	61(18.5)	269(81.5)

(CI =95%) X^2^_df1_ = 19.93, *P*=<0.001

**Table 2:** Significant risk factors for lower urinary tract symptoms among girls

Significant risk factors for girls	LUTS present	LUTS absent	*P* value
a) Anal washing technique			
Proper	8	24 = 32	X^2^=5.183, *P*<0.05
Improper	15	133 = 148	
Total	23	158 = 180	
b) Menstrual pad used			
Sanitary	5	112 = 117	X^2^=21.69, *P*<0.001
Unsanitary	18	45 = 63	
Total	23	157 = 180	
c) Vaginal discharge			
Present	14	8 =22	X^2^=6.138, *P*<0.02
Absent	9	139 =148	
Total	23	157 =180	
d) Pinworms			
Present	7	16 =23	X^2^=7.37, *P*<0.01
Absent	16	141 =157	
Total	23	157 =180	
e) Nutritional status			
>19BMI	9	107 =116	X^2^=7.37, *P*<0.01
<19BMI	14	50 =64	
Total	23	157 =180	
f) Type of toilets			
Good toilets	7	87 =94	X^2^=50.18, *P*<0.001
Bad toilets	16	70 =86	
Total	23	157 =180	

LUTS: Lower urinary tract symptoms

**Table 3:** Significant risk factors for LUTS among housewive

Significant factors for housewives	LUTS present	LUTS absent	*P* value
a) Anal washing technique			
Proper	6	41 =47	X^2^=16.66,
Improper	16	12 =28	*P*<0.001
Total	22	53 =75	
b) Menstrual pad used			
Sanitary	13	31 =44	X^2^=7.65,
Unsanitary	9	22 =31	*P*<0.01
Total	22	53 =75	
c) Vaginal discharge			
Present	15	12 =27	X^2^=13.99,
Absent	7	41 =48	*P*<0.01
Total	22	53 =75	
e) Nutritional status			
>19 BMI	7	36 =43	X^2^=8.28,
<19 BMI	15	17 =32	*P*<0.01
Total	22	53 =75	
h) Sexually transmitted diseases			
Present	9	5 =14	X^2^=10.14,
Absent	13	48 =61	*P*<0.01
Total	22	53 =75	

LUTS: Lower urinary tract symptoms

**Table 4:** Risk factors for LUTS among working women

Significant risk factors for working women	LUTS present	LUTS absent	*P* value
a) Anal washing technique			
Proper	6	51 = 57	X^2^=17.39,
Improper	10	8 =18	*P*<0.001
Total	16	59 =75	
b) Vaginal discharge			
Present	6	6 =12	X^2^=6.99,
Absent	10	53 =63	*P*<0.01
Total	16	59 =75	
c) Prolonged sitting			
Present	6	6 =12	X^2^=6.99,
Absent	10	53 =63	*P*<0.01
Total	16	59 =75	
d) Type of Toilets			
Good toilets	8	12 =20	X^2^=5.66,
Bad toilets	8	47 =55	*P*<0.01
Total	16	59 =75	
f) Sexually transmitted diseases			
Present	7	7 =14	X^2^=8.42,
Absent	9	52 =61	*P*<0.01
Total	16	59 =75	

LUTS: Lower urinary tract symptoms

**Table 5:** Socioeconomic and quality of life losses due to lower urinary tract symptoms

Losses complained	Girls (%) N=23	Housewives (%) N=22	Working women (%) N = 16	Total (%) N = 61
Personal relationships	5(21.7)	11(50)	12(75)	28(46)
Social integration	7(30.4)	13(59)	10(62.5)	30(49)
Interfamilial relationships	5(21.7)	8(36)	12(75)	25(40)
Leisure opportunities	10(43.5)	16(72)	13(81)	39(64)
Reprimand from parents /superiors/family members	11(47.8)	14(63)	10(62.5)	35(57)
Loss of wages	6(26)	7(32)	11(68.75)	24(39)
Loss of leaves/Sickness absenteeism	12(52)	6(27)	13(81)	31(51)
Work Loss	8(34.8)	8(36)	12(75)	28(45)
Financial difficulties	6(26)	16(72)	14(87.5)	36(59)
Lack of Positive feeling	5(22)	12(54.5)	10(62.5)	27
Lack of General adaptation	7(30)	14(63.6)	12(75)	33
Lack of Sexual satisfaction	0	12(54.5)	11(68.8)	23
Loss of Life chances	9(39)	8(36)	13(81)	30
Lack of Spiritual health	0	15(68)	11(68.8)	26

X^2^_df8_= 17.45, *P*<0.05, quality of life losses are significant

A logistic regression analysis was performed in the groups of adolescent girls, working women and housewives. The independent variables for adolescent girls were improper anal washing technique, unsanitary pad usage, malnutrition and presence of intestinal worms. They were significant in predicting an outcome of LUTS in adolescent girls group. The scores for the factors among adolescent girls are as follows: improper anal wash = 5.659 at df_1_, significant *P*<0.017, vaginal discharge 172.042, *P*<0.000, malnutrition 47.021 *P* < 0.000, Pinworms 181.000 *P*<0.000 and unsanitary pad usage 47.021 *P*< 0.000 (-2 Log likelihood 0.000; Cox and Snell R square 0.533; Nagelkerke R square 1.000). The independent variables like usage of unsanitary pads, IUCD insertion and malnutrition were not contributing to UTI among working women. Pinworms, IUCD and bad toilet usage were not contributing to LUTS among housewives.

## DISCUSSION

Overall prevalence of LUTS of 18.5% in this study is considerable but almost equal to other Indian studies.[10–11] The reasons for this high prevalence in this study appear to be due to improper anal washing technique, using unsanitary pads during menses, malnutrition, vaginal discharge and pinworm infestation. Similar results were also revealed by other studies.[1,5,12] But the factors operating were different for the girls, housewives and working women. Unsanitary pads usage, improper anal washing and malnutrition were the main causes among girls. STDs, poor anal wash, unsanitary menstrual pads and malnutrition are the common ones among housewives. Working women were suffering due to prolonged sitting for more than 6 h, using bad toilets and IUCD insertion. Lazy bladder syndrome may be the reason among women with prolonged sitting occupations.

Lowe[6] and Espuña Pons M[7] tried to assess quality of life led by patients suffering from LUTS . These losses among the three groups were also dissimilar in this study. Girls’ major losses were sickness absenteeism and reprimands from teachers while the housewives lost family life and faced family and financial problems. The working women lost leisure time enjoyment, wages and leaves. Economic losses were considerable among the sufferers here as seen in a German study by Vonberg.[13] Broseta Rico also observed similar difference in the profiles of LUTS, but between young women and menopausal women.[8] As different factors were operating in these three groups, group-specific strategies for each group were used in this study. Singh MM utilized health workers to prevent LUTS.[1] Su *et al*. conducted similar interventions successfully in a specific group of clean room workers.[9]

Impact of intervention to bring changes in negative practices yielded good results. As a whole, almost 74% of the beneficiaries appreciated that the interventions were beneficial to them and changed their bad practices to a large extent. More than half of the beneficiaries are continuing the changed lifestyles. However, the role of hormonal factors was not studied in this study. This might be the reason for the difference between the young women and menopausal women in Broseta's study. More elaborate study on cost-effective analysis of LUTS is warranted.

To conclude, the three female groups were suffering from LUTS due to different reasons. An educational intervention through adolescent girls to bring about the reduction in risk factors was successful. This group-specific approach yielded better results.

## APPENDIX-1

### LUTS questionnaire

#### Epidemiological differences of LUTS among female subpopulations

##### PART - A (General Information)

Name: _______________   Age/ ____________________

Address: __________________________________________________

   __________________________________________________

Height: _____ cm     Weight: _____kg; BMI_____

Religion: Hindu / Muslim / Others (Specify)

Monthly income (Rs) __________

Educational status: _illiterate /primary/secondary/higher/professional (tick)

Occupation of father/husband if not working: __________

Occupation details if working:

- Total service: _____Months / years
- Day shift / Night shift / Others (Specify)
- Type of work: Professional / Technical / Unskilled / Others (Specify)

Marital status: Married / Unmarried / Divorce

Duration of married life: _______________

Social class: - Upper/middle/lower

##### PART-B) Case definition for LUTS

Are you having any of these problems?

1. Burning during micturition : Yes / No
  If yes, duration in days  : __________
2. Strangury / Pain during micturition : Yes / No
  If Yes, duration in days  : __________
3. Increased Frequency of micturition (No. of times per day): __________
4. Urgency for micturition: yes/no
5. H / of dull lower abdominal pain  : Yes / No
  1. If Yes. Frequency/month __________

- Periodicity of LUTS:- Periodic / Non-periodic
- Since how many years you are suffering from the above LUTS: 2 / 2 - 5 / 5 - 10 / 10+
- How many episodes per year on average, you are suffering: 1 / 2 / 3 / 4 / 5 / 5+
  Number of days per each episode: < 3 / 3 - 5 / 5 - 10 / 10+

##### PART C. Causes of LUTS (Tick)

- Post pubertal / Post menstrual / Post coital / With pregnancy / Post menopausal IUCD insertion / Catheter-induced / Traumatic / Unsterile vaginal examination/ History of improper anal wash after toilet in childhood or at present / Unsanitary pads usage

1) Personal Hygiene (Tick the answer):

- Cleaning the genital area with  : Water / Paper / Others (Specify)
- Anal washing technique:

Improper (anus to genitals direction and up and down direction) / Proper (anus to behind)

- Using washed undergarments daily : Yes / No
- H / O of Perineal infection  : Yes / No
    If Yes, details :

2). Menstrual History:

- No. of days of menstruation  : _________
- Types of sanitary pads used  : Cloth / Sanitary napkins / unsterile/others (Specify)
- Is the diaper changed daily  : Yes / No

If No, how many times same diaper is used: _________________________

Why is it reused:

1. ____________________
2. ____________________
3. ____________________

3). Any other associated infections present?

- Sexually transmitted diseases(tick) Syphilis/ gonorrhea/ chancroid/ others (use syndromic approach for diagnosis)
- Worms in stools : Yes / No

Details: _____________________

- Any systemic illness  : Yes / No

If Yes, Specify (e.g.: Diabetes etc.) : ________________

4). Relevant local cultural practices and customs:

-   Dietary practices:
  1. ____________________
  2. ____________________
  3. ____________________

-   Any customs or superstitions : Yes / No

If Yes, Specify : ____________________

##### PART - E (Sickness absenteeism and economic losses Measurement)

Sickness absenteeism

- Total work days lost per year on average:
- Total number of work hours lost on average, due to UTI: _____
- Total earnings lost due to UTI as stated by the individual _____
- Number of leaves lost due to UTI in case of working women: _____
- Suffering: Minimum / Tolerable /Intolerable / Disgusting / Life miserable(Tick)

##### F) Quality of life with LUTS as stated by the individual

**Table:**

Facet of life	Poor	Average	Good
Positive feeling			
General adaptation			
Work satisfaction			
Sexual satisfaction			
Personal relationships			
Social integration			
Financial capability			
Life chances			
Interfamilial relationships			
Leisure opportunities			
Spiritual health			
